# Patients’ Assessment of Chronic Illness Care (PACIC): Validation and Evaluation of PACIC Scale among Patients with Type 2 Diabetes in Hungary

**DOI:** 10.5334/ijic.6010

**Published:** 2022-08-08

**Authors:** Viktória Törő, Zsigmond Kósa, Péter Takács, Róbert Széll, Sándorné Radó, Andrea Árokszállási Szelesné, Adrienn Siket Ujváriné, Attila Sárváry

**Affiliations:** 1 Department of Nursing and Midwifery, Faculty of Health Sciences, University of Debrecen, Nyíregyháza, HU; 2 Doctoral School of Health Sciences, University of Debrecen, Debrecen, HU; 3 Department of Health Visitor Methodology and Prevention, Faculty of Health, University of Debrecen, Debrecen, HU; 4 Department of Health Informatics, Faculty of Health Sciences, University of Debrecen, Nyíregyháza, HU; 5 Department of Theoretical and Integrative Health Sciences, Faculty of Health Sciences, University of Debrecen, Nyíregyháza, HU; 6 Department of Emergency and Oxiology, Faculty of Health Sciences, University of Debrecen, Nyíregyháza, HU; 7 Department of Integrative Health Sciences, Faculty of Health Sciences, University of Debrecen, Nyíregyháza, HU

**Keywords:** chronic care model, primary care, PACIC, patient assessment, quality assessment of chronic illness care, Type 2 diabetes mellitus, validation

## Abstract

**Introduction:**

The aims of this study were to evaluate the psychometric properties of the Hungarian translation of the PACIC in a sample of patients with type 2 diabetes and to reveal the associations between the mean PACIC scores and the number of chronic diseases, or visits to GPs, and specialist. An exploratory factor analysis (EFA) has also been performed to test the structural validity of the PACIC scale.

**Methods:**

The Hungarian version of PACIC was validated using randomly selected patients with type 2 diabetes (N = 684) from licensed GP practices.

**Results:**

Floor (1.6%–30.2%) and ceiling effects (11.3–33.6%) were similar of the PACIC scale. The internal consistency of the total scale (Cronbach’s alpha 0.93) was excellent and subscales were good (between 0.73–0.9). The mean scores of each PACIC subscale group were between 2.99–3.53. There was a weak significant correlation between the mean PACIC scores of subscales and the number of GP visits (p < 0.001), and specialist visits (p < 0.001). The EFA identified four factors on the sample (KMO = 0.931). Gender and education showed correlation with some new factors.

**Conclusion:**

The psychometric properties of the Hungarian version of PACIC questionnaire showed a reasonable level of validity among patients with type 2 diabetes. Now, this instrument is ready to assess the chronic care of diabetic patients in Hungary.

## Introduction

Noncommunicable diseases especially cardiovascular, cancer, respiratory diseases and diabetes are the leading causes of death both worldwide and in Europe [1,2]. According to the estimation of the International Diabetes Federation (IDF) in 2017 all over the world the number of people suffering from diabetes were about 425 million the prevalence of disease was 8.8%. In the European Region 58 million patients were registered and the age-adjusted prevalence was 6.8% [3].

The leading causes of death in Hungary, similarly to the developed countries were the cardiovascular and cancer diseases in 2016 [4]. The raw prevalence of diabetes was 9.5% and the proportion of diabetes caused deaths of people under 60 years was 25% [5].

Literature mentions different chronic disease models. One of the best known and widely used model is the chronic care model (CCM) [6]. The CCM is a comprehensive model aiming to improve the patient-centered, evidence-based care of chronically ill patients [7]. The Assessment of Chronic Illness Care has been developed to measure deficiencies, strenghts and weaknesses of CCM from the perspective of clinicians [8].

The quality care of chronic patients on one hand is measured by objective parameters (blood pressure, haemoglobin A1c, lipid parameters), on the other hand patients’ perception about care can be measured by questionnaires, which is another important, subjective aspect of the quality of care. The Patient Assessment of Chronic Illness Care (PACIC) was constructed and used to assesses how much the provided care is congruent with the Chronic Care Model [9]. The evaluation was carried out in different countries, and in different patient groups, typically suffering from high prevalent chronic diseases. PACIC was delivered among others patients with diabetes, chronic pulmonary obstructive diseases, but other patient groups also were involved in these types of studies [10,11,12,13,14,15,16]. The PACIC scale was validated and succesfully used among diabetic patients in some European countries to assess the chronic care managements of these patients [17,18,19,20]. However, several issues arose during the evalution of the results i.e. the need to improve of some items, problems related to variablitity, factor-structure of the scale and the comparison of the results between countries [15,20,21,22].

The Hungarian version of PACIC scale was developed in 2013 and it was applied among people living in Roma colonies suffering from chronic diseases but it was not validated [23].

The first aim of this study was to evaluate the psychometric properties of the Hungarian translation of the PACIC in a sample of patients with type 2 diabetes. The second aim of the study was to deeper reveal the associations between the mean PACIC scores and the number of chronic diseases, or visits to GPs, and specialist and to test the structural validity of the Hungarian translation of the PACIC scale.

## Methods

### Study design and settings

The current nationwide survey was conducted in primary care in 2018 among registered patients suffering from diabetes type 2. There was a two-stage sampling procedure: the first step was to randomly select licensed Hungarian GPs’ practices on the basis of official records, the second step was to randomly select certain individuals from a pool of diabetic patients arranged in order of birth. So finally, 20 GPs’ practices from 7 regions were selected for this cross-sectional study and the target population included 800 diabetic patients.

Criteria of selection:

– patients over the age of 18,
– patients diagnosed with diabetes type 2 for at least 6 months,
– patients belonging to a certain GP’s practice for at least 6 months.

Community nurses working with the general practitioners (GPs) delivered the questionnaires to the patients with chronic diabetes. Written informed consent was obtained from participants. The doctor and nurses were preliminarily presented with the research plan and the survey’s aims. Anonymity was emphasized so that the personality of the GP would not influence the response. The patients put the completed questionnaires into a closed collecting box placed in the waiting-room.

### Measures

The Hungarian version of the original PACIC questionnaire (20 items form) was supplemented with demographic- (age, gender, marrital status, education, place of living) and disease-related questions. Patients were asked to provide self report of the chronic diseases, long-term conditions from a list and the number of GPs and specialists they visited during the last 6 months. The 20 items of questions for examining the quality of patient care were grouped according to the original study [9] into 5 topics: patient activation (items 1–3); delivery system design/decision support (items 4–6); goal setting/tailoring (items 7–11); problem-solving/contextual counselling (items 12–15); follow-up/coordination (items 16–20). In case of each question the patients grade the quality of care they received in the last 6 months within the primary care on a scale from 1 (never) up to 5 (always). Evaluation is made by averaging the scores given to the various elements, hereinafter referred to as the average of the PACIC scores.

The Hungarian translation of the self-completing PACIC questionnaire used in this survey was developed in 2013, based on the guidelines of the WHO [23,24].

### Validation of the questionnaire

The validity of the Hungarian PACIC questionnaire was tested for the following psychometric properties: content validity, internal consistency reliability, convergent and construct validity. Descriptive data on predetermined subscale and total scale levels were also presented.

### Acceptability

The acceptability of the translated items were assessed by exploring rates of missing data on item level. The researchers also calculated the proportion of the respondents with the lowest (floor effect) and the highest (ceiling effect) possible scores on PACIC scale at item level and original predetermined subscales in order to prove the acceptability of the instrument. The floor and ceiling effects were measured as the percent of patients who reported a minimum (i.e., 1) or maximum (i.e., 5) scores. If a substantial proportion of the respondents score at either extreme of range, suggesting that the scale is not sensitive to measure the real differences [25]. Frequency less than 30% was accepted [26]. A stricter criterion was used on the total PACIC scale (<1.5 or >4.5).

### Reliability

The internal consistency of the questionnaire was assessed by calculating the Cronbach’s α value both subscales and total scale levels. Good internal consistency is needed to justify summarizing of items at both levels [27]. Cronbach’s alpha value between 0.70 and 0.80 can be considered acceptable and scores over 0.80 as excellent [25], however, alphas should not exceed 0.95 [27]. The inter-correlations between the predetermined subscales were assessed with Spearman’s rho.

Association analyses (related to demographic characteristics and number of chronic conditions and number of visits) were performed by independent sample of t-test, ANOVA, Kruskal-Wallis or Mann-Whitney U tests, Sperman’s rho – rank correlation coefficient, as appropriate.

Exploratory factor analysis was performed on the PACIC instrument to explore the latent feature of structure of 20 item scale. Tests of sampling adequacy (Kaiser–Meyer–Olkin-criterion ≥0.50) and multicollinearity (Bartlett test of sphericity with a P-value < 0.05) were undertaken prior to factor extraction to ensure that the scale items were appropriate for principle component analysis. The EFA produced solutions from one to six factors. Calculation results were measured using multiple fit indices. The degree of fit was evaluated using χ^2^ test (degree of freedom df, associated p value); comparative fit index (CFI, Hull method); Tucker-Lewis index (TLI; >0.95 very good, >0.90 good). It was even used root-mean-square error of approximation (RMSEA; 0.06> very good; >0.08 good).

SPSS and R statistical programmes (version 22.0 and version 4.02) were used for data recording and analysis.

### Ethical approval

The study was approved by the Hungarian Medical Research Council.

## Results

A total of 684 questionnaires were returned from the 800 questionnaires (response rate: 85.5%) distributed among diabetic patients, all of them were evaluable.

The mean age of the respondents was 63.19 (SD = 12.79), 51.6% of them were female. The main sociodemographic characteristics have been shown in Table 1.

**Table 1:** Patients’ main characteristics.


CHARACTERISCTICS	N (%) (N = 684)

**Gender**	

male	331 (48.4)

female	353 (51.6)

**Age** (min 19, max 96)	

≤54	138 (20.2)

55–64	206 (30.1)

65+	340 (49.7)

**Marital status**	

married	401 (58.6)

widow	151 (22.1)

single	53 (7.8)

divorced	70 (10.2)

other	9 (1.3)

**Education**	

primary school or less	169 (24.7)

secondary school/secondary grammar school	395 (57.8)

higher education	120 (17.5)


Most patients who filled in the questionnaire suffered from other chronic diseases besides diabetes. Hypertension had the highest prevalence (74.7%), but the prevalence of arthritis (36.1%) and chronic pain (22.8%) were also high among others. The prevalence of depression (13.9%) and ischaemic heart diseases (13.5%) was similar among respondents.

12.5% of the respondents did not have any other diagnosed chronic diseases besides diabetes, 28.2% had one and 13.4% suffered from four or more chronic diseases (2 chronic diseases 28.7%; 3 chronic diseases 18.9%). During the last six months, 10.5% of the patients visited their GP once, 39.0% of them 2–3 times, and 50.4% 4 or more than 4 times. Regarding the number of specialists visits, they are much less. 48.6% of the patients attended a specialist appointment once and 36.0% 2–3 times in the last six months (4–5 × 10.4%; ≥6 × 5.1%).

The response rate was high with only two missing items in 2 respondents’ questionnaire. The 20 questions on assessment of the quality of care can be subscaled into 5 topics (Patient activation; Delivery system design/decision support; Goal setting; Problem-solving/contextual counseling; Follow-up/coordination).

Regarding the results of quality-of-care responses, 41.5% of the respondents (rated 4 or 5 of the first item) cooperated with their GPs to develop a treatment plan for their chronic disease, according to 40.4% of them doctors and nurses always considered their values, belief and traditions respectively when they proposed treatment. More than sixty percent (62.0%) of respondents were asked about their problems related to taking medicine at every attended appointment. 65.5% of the patients were satisfied completely with the care of their GPs, these patients’ opinion was that the whole procedure of care was almost always well organized.

Patients’ opinion was examined about the extent of personalization of their care. Forty percent of respondents (40.1%) reported they had never been asked about their health behavior in any way, 43.4% had never been recommended for group work that could help them to deal with their chronic disease, to get well, or to change their lifestyle. 56.1% of patients were referred to a dietitian, patient education and counseling specialist in almost all cases.

Floor and ceiling effects showed a wide variation at single items level, but it was low both on the subscales and on the total PACIC scale. Similar floor and ceiling effects were found in our study. The floor effects ranged between 1.6% and 30.2% (>30% for one item), while the ceiling effects ranged between 11.3% and 33.6% (>30% for two items). Item 9 (“Given a copy of my treatment plan.”) showed the highest floor effect, while the ceiling effect was more than 30% for item 5 (“Satisfied that my care was well organized.”) and item 20 (“Asked how my visits with other doctors were going.”). On subscales the highest value (ceiling effect) was 10.1% for *Follow-up/coordination*, however there was no floor/ceiling value, which exceeded the 20% limit on subscale level (Table 2). Based on the responses of the quality survey of care, the mean total PACIC score was 3.24 (SD 0.85). The total PACIC scale approched the normal distribution; however, it was moderately skewed (skewness 0.530, kurtosis – 0.248). The five subscales means moved in a narrow range, ranged from 2.99 (1.02) for *Goal setting/tailoring* to 3.53 (0.93) for *Delivery system design/decision support*.

**Table 2:** Descriptive data on PACIC scale (N = 684).


	MEAN (SD)	FLOOR EFFECT^a^	CEILING EFFECT^a^

		N (%)	

Patient activation (1–3 items; no missing data)	3.32 (0.99)	9 (1.3)	50 (7.3)

Q1	3.17 (1.18)	64 (9.4)	98 (14.3)

Q2	3.08 (1.19)	75 (11.0)	87 (12.7)

Q3	3.71 (1.08)	21 (3.1)	185 (27.1)

Delivery system design/decision support (4–6 items; no missing data)	3.53 (0.93)	2 (0.3)	65 (9.5)

Q4	3.05 (1.34)	118 (17.3)	116 (17.0)

Q5	3.85 (1.04)	11 (1.6)	225 (32.9)

Q6	3.68 (1.07)	21 (3.1)	169 (24.7)

Goal setting/tailoring (7–11 items; 1 missing item in 1 respondent’s questionnaire)	2.99 (1.02)	7 (1.02)	35 (5.12)

Q7	3.24 (1.22)	80 (11.7)	107 (15.6)

Q8	3.23 (1.19)	62 (9.06)	114 (16.67)

Q9	2.81 (1.53)	206 (30.2)	143 (20.9)

Q10	2.77 (1.37)	184 (26.9)	77 (11.3)

Q11	2.91 (1.29)	128 (18.7)	77 (11.3)

Problem-solving/contextual counselling (12–15 items; 1 missing item in 1 respondent’s questionnaire)	3.23 (1.02)	8 (1.2)	48 (7.0)

Q12	3.00 (1.38)	144 (21.1)	115 (16.8)

Q13	3.13 (1.25)	86 (12.6)	109 (15.9)

Q14	3.40 (1.20)	56 (8.2)	136 (19.9)

Q15	3.40 (1.20)	56 (8.2)	134 (19.6)

Follow-up/coordination (16–20 items; no missing data occured)	3.29 (1.01)	5 (0.7)	69 (10.1)

Q16	2.94 (1.48)	180 (26.4)	136 (19.9)

Q17	2.82 (1.40)	183 (26.8)	92 (13.5)

Q18	3.48 (1.27)	72 (10.5)	169 (24.7)

Q19	3.52 (1.29)	63 (9.2)	199 (29.1)

Q20	3.70 (1.23)	48 (7.0)	230 (33.6)

**PACIC total score (20 items; 2 missing items alowed)**	3.24 (0.85)	0 (0)	5 (0.73)


^a^ Floor and ceiling effects = percent of respondents attaining minimum or maximum scores (1/5).

In terms of reliability the Cronbach’s α value for the whole scale was 0.936, while the Cronbach’s α value for the subgroup was as follows: patient activation 0.818 (3 items), delivery system design/decision support 0.730 (3 items), goal setting/tailoring 0.823 (5 items), problem solving/contextual 0.830 (4 items) and follow-up/coordination 0.815 (5 items).

The inter-correlation (Spearman’s rho) between the subscales was moderate to high, being the highest between the Problem-solving and Goal-setting scales (0.752; p < 0.001) and Goal-setting and Decision-support scales (0.660; p < 0.001), whereas the Follow-up scale was the least correlated with the other scales, and the lowest with the Patient-activation scale (0.489; p < 0.001). The Goal-setting (0.881; p < 0.001) and Problem-solving (0.892; p < 0.001) scales correlated the highest with the total PACIC scale and the Follow-up scale the least (0.725; p < 0.001).

The number of diseases and the age showed a moderately weak relationship (Pearson’s r = 0.314, p < 0.001). Examining the relationship between the number of diseases and PACIC mean score, significant relationship was not found. However, as the number of diseases increased, the number of attended appointments at GPs and specialists increased paralelly (Pearson’s r = 0.208 and r = 0.170, p < 0.001 in both cases).

There was a weak significant association between the mean PACIC scores of subscales and the number of visits to GPs (the value of Spearman’s rhos respectively were 0.044 (p = 0.25); 0.157 (p < 0.001); 0.127 (p < 0.001); 0.122 (p < 0.001); 0.128 (p < 0.001)). There was a weak significant association between the mean PACIC scores subscales and visits to specialists (the value of Spearman’s rhos respectively were 0.168 (p < 0.001); 0.127 (p = 0.001); 0.151 (p < 0.001); 0.178 (p < 0.001); 0.121 (p = 0.002)).

The means of the subscales for the numbers of the visits of GPs and specialist are shown in Table 3. In all cases, there was a significantly higher PACIC subscale mean in the group with more than 6 visits.

**Table 3:** The numbers of visits of GPs and specialist and mean PACIC scores.


NUMBER OF GP VISITS IN THE LAST 6 MONTHS	PATIENT ACTIVATION (MEAN (SD))	DELIVERY SYSTEM DESIGN/DECISION SUPPORT (MEAN (SD))	GOAL SETTING (MEAN (SD))	PROBLEM-SOLVING/CONTEXTUAL COUNSELLING (MEAN (SD))	PROBLEM-SOLVING/CONTEXTUAL COUNSELLING (MEAN (SD))

**1x**	3.31 (1.04)	3.38 (0.99)	2.80 (1.11)	3.06 (1.17)	3.15 (1.03)

**2–3x**	3.33 (0.99)	3.46 (0.89)	2.92 (0.93)	3.18 (0.98)	3.24 (0.92)

**4–5x**	3.16 (0.92)	3.39 (0.94)	2.92 (0.96)	3.14 (1.00)	3.19 (1.00)

**≥6**	**3.50 (1.01)**	**3.86 (0.90)**	**3.28 (1.12)**	**3.51 (1.01)**	**3.57 (1.11)**

*p	0.017	0.000	0.000	0.001	0.001

**NUMBER OF SPECIALIST VISITS IN THE LAST 6 MONTHS**	**PATIENT ACTIVATION (MEAN (SD))**	**DELIVERY SYSTEM DESIGN/DECISION SUPPORT (MEAN (SD))**	**GOAL SETTING (MEAN (SD))**	**PROBLEM-SOLVING/CONTEXTUAL COUNSELLING (MEAN (SD))**	**PROBLEM-SOLVING/CONTEXTUAL COUNSELLING (MEAN (SD))**

**1x**	3.15 (1.03)	3.43 (0.95)	2.85 (0.98)	3.09 (1.00)	3.19 (0.98)

**2–3x**	3.43 (0.94)	3.51 (0.89)	3.00 (1.01)	3.28 (1.00)	3.26 (1.00)

**4–5x**	3.41 (0.92)	3.69 (1.02)	3.21 (1.06)	3.44 (1.07)	3.35 (1.09)

**≥6**	**3.84 (0.72)**	**4.02 (0.73)**	**3.73 (1.02)**	**3.93 (0.91)**	**4.00 (1.06)**

*p	0.000	0.002	0.000	0.000	0.000


* ANOVA test. The highest mean PACIC scores are shown in bold. These mean values are significantly higher than the other group means.

The analysis of the different demographic groups has not shown significant difference between mean PACIC scores (gender, age, education, marital status) (Table 4).

**Table 4:** Equality between mean PACIC scores and patients’ demographic characteristics (N = 684).


CHARACTERISTIC	PACIC MEAN (SD)	P-VALUE

**Gender**		

male	3.24 (0.82)	0.983^a^

female	3.24 (0.88)	

**Age**		

≤54	3.27 (0.87)	0.597^b^

55–64	3.28 (0.88)	

65+	3.21 (0.83)	

**Professional education**		

upper secondary education or less	3.24 (0.85)	0.616^a^

higher education	3.28 (0.88)	

**Marital status**		

married	3.23 (0.86)	0.805^b^

widow	3.25 (0.87)	

single	3.32 (0.77)	

divorced	3.30 (0.84)	


^a^ Independent samples t-test. ^b^ ANOVA.

### Exploratory factor analysis

The associations between the 20 questions of quality of care were analyzed by exploratory factor analysis (EFAPromax rotation).

The EFA produced solutions from one to six factors. The results are shown in Table 5. All goodness-of-fit incidices show correct results model (KMO = 0.931; Bartlett test p = 0.000). These results and the Hull method (based on comparative fit index CFI, Velicer analysis) proposed a four-factor.

**Table 5:** Exploratory factor analysis goodness-of-fit results (1–6 factors; N = 684).


FACTORS	χ^2^	DF	P	CFI	TLI	RMSEA

1	1798.8	170	<1.1e–26	0.9714	0.718	0.132

2	922.53	151	<5.7e–11	0.9879	0.794	0.113

3	508.7	133	2.5e–45	0.9952	0.832	0.102

**4**	**277.82**	**116**	**2.7e–15**	**0.9991**	**0.878**	**0.087**

5	176.94	100	3.3e–06	–	0.901	0.078

6	98.66	85	<0.15	–	0.923	0.069


Tucker-Lewis index (TLI; >0.95 very good, >0.90 good). Root-Mean-Square Error of Approximation (RMSEA; 0.06> very good; >0.08 good).

**Figure 1:**
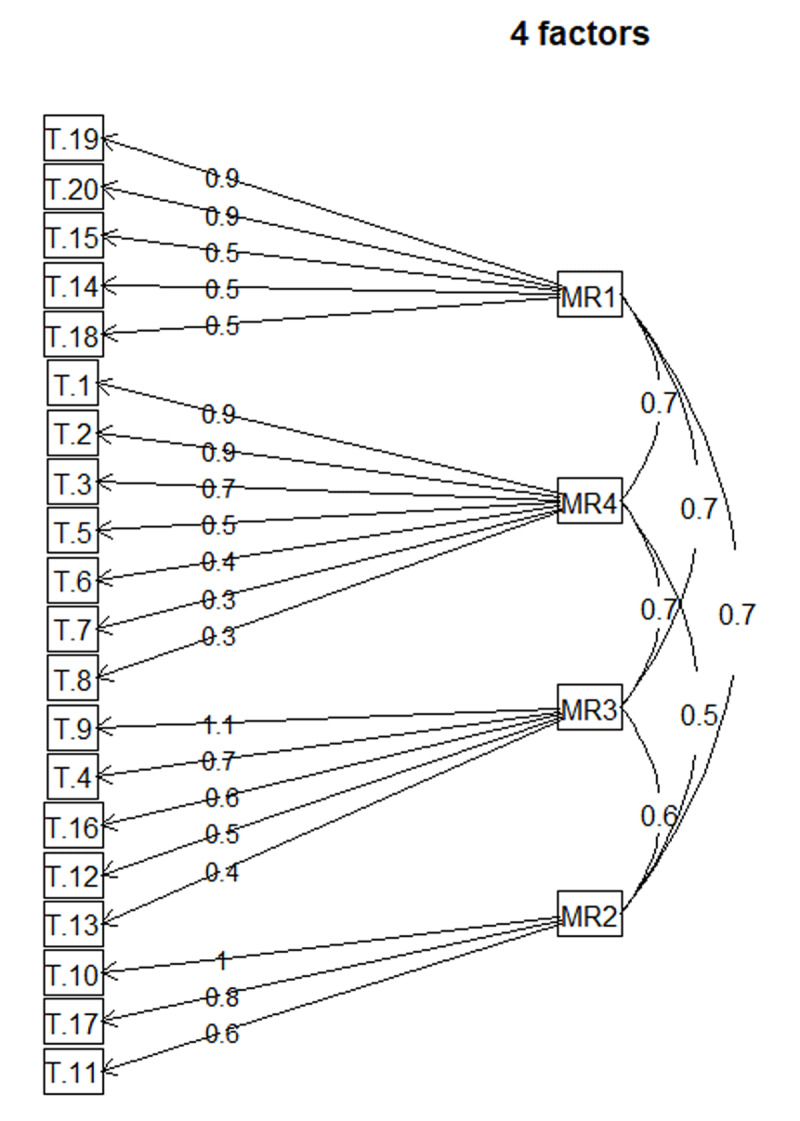
Factor Analysis – four-factor model. Standardized loadings (pattern matrix) based upon correlation matrix. The figure also indicates interactions.

The factor loading values of the four-factor model are shown in Table 6. The grouping of items is shown in Figure 1.

**Table 6:** Factor Analysis: using method = minres; rotation “promax”. Standardized loadings (pattern matrix) based upon correlation matrix.


PREDETERMINED SUBSCALES AND ITEMS	F1 DETERMINE PURPOSES MR4	F2 INVOLVEMENT OF SPECIALISTS MR1	F3 ENCOURAGING PATIENT ACTIVITY MR2	F4 PERSONALIZATION MR3

Patient activation				

1. Asked for my ideas when we made a treatment plan	**0.94**	–0.16	0.00	–0.09

2. Give choices about treatment to think about.	**0.90**	–0.16	0.04	–0.10

3. Asked to talk about any problems with my medicines or their effects.	**0.71**	0.20	–0.02	–0.11

4. Given a written list of things I should do to improve my health.	0.15	–0.06	–0.09	**0.68**

5. Satisfied that my care was well organized.	**0.54**	0.36	–0.23	0.03

6. Shown how what I did to take care of myself influenced my condition.	**0.41**	0.22	–0.05	0.23

7. Asked to talk about my goals in caring for my condition.	**0.31**	0.11	0.25	0.21

8. Helped to set specific goals to improve my eating or exercise.	**0.31**	0.11	0.14	0.30

9. Given a copy of my treatment plan.	0–0.17	0.24	–0.03	**1.07**

10. Encouraged to go to a specific group or class to help me cope with my chronic condition.	–0.02	–0.12	**1.02**	–0.05

11. Asked questions, either directly or on a survey, about my health habits.	0.15	–0.12	**0.62**	0.21

12. Sure that my doctor or nurse thought about my values, beliefs, and traditions when they recommended treatments to me.	–0.02	–0.03	0.22	**0.49**

13. Helped to make a treatment plan that I could carry out in my daily life.	0.04	0.26	0.13	**0.44**

14. Helped to plan ahead so I could take care of my condition even in hard times.	0.11	**0.50**	0.12	0.14

15. Asked how my chronic condition affects my life.	0.16	**0.51**	0.17	0.03

16. Contacted after a visit to see how things were going.	0.15	0.36	–0.15	**0.62**

17. Encouraged to attend program sin the community that could help me.	–0.16	0.31	**0.81**	–0.16

18. Reffered to a dietitian, health educator, or counselor.	–0.06	**0.49**	0.30	–0.05

19. Told how my visits with other types of doctors, like an eye doctor or other specialist, helped my treatment.	–0.07	**0.95**	0.06	–0.17

20. Asked how my visits with other doctors were going.	–0.03	**0.87**	–0.09	–0.07


The given names of these generated groups reflect the local Hungarian circumstances.

Factor 1 (MR4) was called ‘Self-management’. There were 7 items of the original 20 questions in this group (1; 2; 3; 5; 6; 7; 8) coming from different subscale topic groups: all the questions of *Patient activation* (1; 2; 3) and 2–2 questions from *Delivery system design* and *Goal setting* groups (5; 6; 7; 8).

Factor 2 (MR1) was named ‘Involvement of Specialists’. This referred to question informing the researchers about what specialists the patients were referred to by primary care, and – after consultation with specialists – the GP could provide assistance in how the patient could or could not adapt this information to his or her own life.

Factor 3 (MR2) was named ‘Encouraging Patient Activity’. This includes questions (10; 11; 17) that provides information about patient satisfaction with community programs and group activities recommended by the GP.

Factor 4 (MR4) was named ‘Personalization’. These questions examine the personalization of the treatment plan based on cooperation between the GP and the patient. The answers informed the researchers whether the treatment plan was prepared considering the patient’s belief and values. Moreover, the extent of help in adapting the treatment plan to patient’s everyday life can also be estimated. Thus, a clearer view can be obtained about care and patient follow-up in primary care.

The new variables formed by factor analysis were further examined. The new factors showed no significant correlation with age and disease number (correlation analysis). Based on independent samples t-test Factors 1, 2, 3 did not differ by gender (p = 0.977; p = 0.175; p = 0.99), only factor 4 differed by gender (p = 0.003). The mean for the female group was significantly higher.

Factor 1 showed a significant difference between the different educational groups (ANOVA, p = 0.029). Lower educational attainment showed lower goal setting. A high level of education means more conscious treatment of the disease.

Factor 2 also showed a significant difference between the different educational groups (ANOVA, p = 0.048). Those with lowest and highest qualifications involve specialists in treatment the least. The most acceptance of the help is in high school graduates. For factors 3 and 4, there was no significant difference by educational attainment.

## Discussion

The main aim of this study was to validate the Hungarian version of the PACIC scale and evaluate the chronic care management of patients with type 2 diabetes at primary care level. The response rate was high in this study and the unrespondent rate to the items was minimal. The validation analysis showed a good acceptability and internal consistency reliability (the Cronbach’s α value for the subscales were more than 0.800, except the subscale 2) of the original instrument in our sample. The total PACIC scale approched the normal distribution. Regarding the acceptability we found only three items where the floor or ceiling effects exceded the 30% limit and there was no subscale where the floor or ceiling effects exceded the more stricter 20% limit. The results (similar floor and ceiling effect) are in line with the previous findings of Kim et al (2021), while others found a more notably floor effects [17,28,29,30]. It is important to note, that in our study the exploratory factor analysis identified four-factor structure as best fitting model, while the five-factor model also showed a good goodness of fit results.

The background data of patients showed that the most prevalent chronic diseases and conditions among patients were hypertension, arthritis and chronic pain. Results showed that about 40% of the respondents cooperated with their GPs to develop a treatment plan for their chronic disease and reported that doctors and nurses always considered their values, belief and traditions respectively when they proposed a treatment. More than sixty percent of respondents were asked about their problems related to taking medicine and the majority of the patients were completely satisfied with the care provided by their GPs.

Taking the PACIC subscale scores the mean scores were around 3.2, the *Delivery system design/decision support* subgroup was rated highest, while the *Goal setting* received the lowest mean score. Some studies showed lower total and subscale scores [17,30], while others found similar scores [9,28,31]. Comparing our results with the findings of the latest study carried out among patients with type 2 diabetes in Finland published in 2018 [17] our PACIC scores were higher in each subgroup. In both studies the *Delivery system design/decision support* received the greatest score. However, in the Finnish study the respondents rated the *Follow up/coordination* subgroup with the lowest score. The reasons of the difference among studies can be explained by the different health care systems operating in the countries, the different characteristics (age, gender ethnic groups, diseases conditions) and expectations toward the health care system of patients.

Similarly to the previous studies the association between the PACIC scores and the sociodemographic characteristics of respondents were investigated. Drewes and colleagues found negative association between PACIC scores and age and education levels [20]. However, they did not find association with gender, duration of diabetes and comorbidity [20]. Simonsen and colleagues revealed negative association between PACIC scores and gender (females had lower score), age, marital status and duration of diabetes, and did not find association with education [17]. We have not found associations between PACIC mean score and demographic characteristics of patients as well as the number of diseases. The difference can be explained by the relatively smaller study sample and it has to note that the literature is inconsistence regarding associations between mean PACIC scores and demographic variables. However, there was a significant association between the mean PACIC scores of subscales and the number of visits to GPs and specialists. Simonsen et al (2018) also found positive association between PACIC scores and the continuity of care [17]. It is reasonable to assume that patients, who meet their phycisians more regularly, are more satisfied with the performance of the health care system and the quality of care.

The exploratory factor analysis identified four factors in our sample, which was different from the original five subscales. They were named as ‘Self-management’, ‘Involvement of Specialists’, ‘Encouraging Patient Activity’, and ‘Personalization’. Our identified factors are relatively similar to the three factors found by Simonsen et al. [17] The items of the first two factors are mainly overlapping, while our third and fourth factors and Simonsen’s third factor consist of some different items. The subscales of the original version of the instrument developed by Glasgow and colleagues were analyzed by confirmatory factor analysis and showed a moderate goodness of the fit for the overall modell [9]. Iglesias et al (2014) performed a comprehensive statistical analysis to evaluate the five-dimensional model of Glasgow. They found that a single-dimension model considering 11 out of the 20 PACIC items model showed the best fit to their sample and concluded that a single-dimension structure comprised of all 20, or a subset of 11 items should be used [22]. The subsequent studies conducted in different populations and patient groups found different number of factors by exploratory factor analysis. Two studies [15,32] have found and used one factor in the analysis, similar to Iglesias. An Australian research group [33] and some European studies identified 2 dimensions [13,34], while others found 3–5 dimension-structure of PACIC instrument [17,35,36]. The discrepancies between the dimension-structure of these studies may be explained by the differences between the applied methology approach, the health care system, the patients’ interaction with the health care system, and the sample of the population. Fan and colleagues argued that also the sample size and the patients’ awareness at least partially by the CCM principles can contribute the number of factors [36]. However, in case of our study it can be dassume that the patients at primary care level did not receive information about this model.

The correlation analysis with the new factors showed no significant correlation with the age and disease number and only factor 4 revealed significant connection with gender (females had higher score). Lower educational attainment was found to have lower score for goal setting (determine purposes) and the lowest and highest qualification groups demanded involvement of specialists in treatment the least. These results can be explained by the previous findings that there is a strong correlation between health literacy or concious health behaviour and educational level [37,38,39].

### Strengths and limitations

The strength of this study was the high response rate (85.5%) and the randomized sampling method.

The limitations of the study could be that not each of the questions were fully understandable for the patients. The social desirability may have influenced the responses. It cannot be excluded that the distribution method of the questionnaire (community nurses handed them to the patients) influenced the scores of respondents. We did not perform test-retest and evaluate reproducibility of the scale. The researchers tried to limit the GP’s attitude to influence the respondent’s score as the results were not reported to them individually. We could not exclude the possibility that the nurses helped the respondents to understand the questions if they had problems with it.

## Conclusions

In this study the psychometric properties of the Hungarian version of PACIC questionnaire showed a reasonable level of validity. In line with the previous findings, this study confirmes that the Hungarian version of PACIC scale is a valuable, reliable, and useful instrument to assess the chronic care of diabetic patients at primary care level. However, the exploratory factor analysis identified four dimensions in our study population therefore this analysis recommended to use prior to data analysis of other patient populations. In Hungary the patients are moderately satisfied with their care in the primary care level. This finding raises attention for improving and strengthening the quality of care of diabetic patients at primary care level.
